# Kinetics of antibody responses to PfRH5-complex antigens in Ghanaian children with *Plasmodium falciparum *malaria

**DOI:** 10.1371/journal.pone.0198371

**Published:** 2018-06-08

**Authors:** Frederica D. Partey, Filip C. Castberg, Edem W. Sarbah, Sarah E. Silk, Gordon A. Awandare, Simon J. Draper, Nicholas Opoku, Margaret Kweku, Michael F. Ofori, Lars Hviid, Lea Barfod

**Affiliations:** 1 Noguchi Memorial Institute for Medical Research, University of Ghana, Legon, Ghana; 2 Centre for Medical Parasitology at Department of Immunology and Microbiology (ISIM), Faculty of Health and Medical Sciences, University of Copenhagen, Copenhagen, Denmark; 3 Department of Infectious Diseases Copenhagen, University Hospital (Rigshospitalet), Copenhagen, Denmark; 4 Department of Clinical Microbiology, Copenhagen University Hospital (Rigshospitalet), Copenhagen, Denmark; 5 The Jenner Institute, University of Oxford, Oxford, United Kingdom; 6 West Africa Centre for Medical Biology of Infectious Pathogens, Department of Biochemistry, Cell and Molecular Biology, University of Ghana, Legon, Ghana; 7 Hohoe Municipal Hospital, Hohoe, Ghana; Universidade Federal de Minas Gerais, BRAZIL

## Abstract

*Plasmodium falciparum* PfRH5 protein binds Ripr, CyRPA and Pf113 to form a complex that is essential for merozoite invasion of erythrocytes. The inter-genomic conservation of the PfRH5 complex proteins makes them attractive blood stage vaccine candidates. However, little is known about how antibodies to PfRH5, CyRPA and Pf113 are acquired and maintained in naturally exposed populations, and the role of PfRH5 complex proteins in naturally acquired immunity. To provide such data, we studied 206 Ghanaian children between the ages of 1–12 years, who were symptomatic, asymptomatic or aparasitemic and healthy. Plasma levels of antigen-specific IgG and IgG subclasses were measured by ELISA at several time points during acute disease and convalescence. On the day of admission with acute *P*. *falciparum* malaria, the prevalence of antibodies to PfRH5-complex proteins was low compared to other merozoite antigens (EBA175, GLURP-R0 and GLURP-R2). At convalescence, the levels of RH5-complex-specific IgG were reduced, with the decay of PfRH5-specific IgG being slower than the decay of IgG specific for CyRPA and Pf113. No correlation between IgG levels and protection against *P*. *falciparum* malaria was observed for any of the PfRH5 complex proteins. From this we conclude that specific IgG was induced against proteins from the PfRH5-complex during acute *P*. *falciparum* malaria, but the prevalence was low and the IgG levels decayed rapidly after treatment. These data indicate that the levels of IgG specific for PfRH5-complex proteins in natural infections in Ghanaian children were markers of recent exposure only.

## Introduction

*P*. *falciparum* malaria is estimated to cost more than half a million lives every year, mainly in tropical Africa [1]. The disease burden is highly concentrated among young children, because survivors gradually acquire protective immunity in response to repeated infection [2]. Protection acquired this way is notoriously sluggish to develop, is incomplete, and has limited durability. These characteristics have mainly been related to the extensive polymorphism and antigenic variation in the parasite’s asexual blood-stage antigens that are the key targets of naturally acquired immunity to the disease. Many consider these features as insurmountable obstacles to the development of vaccines targeting this part of the parasite life cycle, but the recent discovery of a set of conserved antigens that appear indispensable for completion of the asexual multiplication cycle has raised new hopes.

The asexual multiplication cycle initiates when a merozoite invades an erythrocyte. Despite the rapidity of invasion, it is a multi-step process that involves numerous parasite molecules, most of which are redundant and polymorphic [3]. However, about ten years ago it became apparent that the reticulocyte-binding protein homolog 5 (PfRH5) is both highly conserved and indispensable for invasion [4, 5]. Since then, much has been learned about the function of PfRH5 in invasion, and several additional parasite molecules that play important roles in it have been identified.

It is now known that the structured domain of PfRH5 (central and C-terminal region) binds to the erythrocyte receptor basigin, thereby forming the contact point that initiates parasite entry [6, 7]. Two other conserved parasite molecules, the cysteine-rich protective antigen (CyRPA) and Pf113 (a.k.a. P113 [8], which also binds to the disordered N-terminus of PfRH5 [9]), are also required for successful invasion [8, 10, 11]. The GPI-anchored Pf113 presumably tethers the otherwise soluble PfRH5/CyRPA complex to the merozoite surface, while CyRPA appears to be required to allow the release of the complex from the merozoite surface by binding yet another parasite antigen, the PfRH5-interacting protein (Ripr), in a way that is incompatible with the interaction of PfRH5 and Pf113 [9, 12, 13]. PfRH5-specific antibodies, including antibodies that target the N-terminus and do not prevent binding of PfRH5 to basigin, as well as antibodies to CyRPA and Ripr, can all prevent successful merozoite invasion [9, 11, 12, 14–16]. These findings point to a crucial role for the PfRH5/CyRPA/Ripr/Pf113 complex in parasite survival and identify them as promising potential vaccine targets [17, 18]. However, only little is known (from a small handful of studies to-date) about the role of these antigens in clinical protection from malaria that is gradually acquired by individuals naturally exposed to *P*. *falciparum* parasites [19–22]. We therefore set out to obtain such information regarding PfRH5, CyRPA, and Pf113 in a cohort of Ghanaian children.

## Results

### Prevalence and levels of IgG specific for PfRH5-complex components and other merozoite antigens

**Fig 1:**
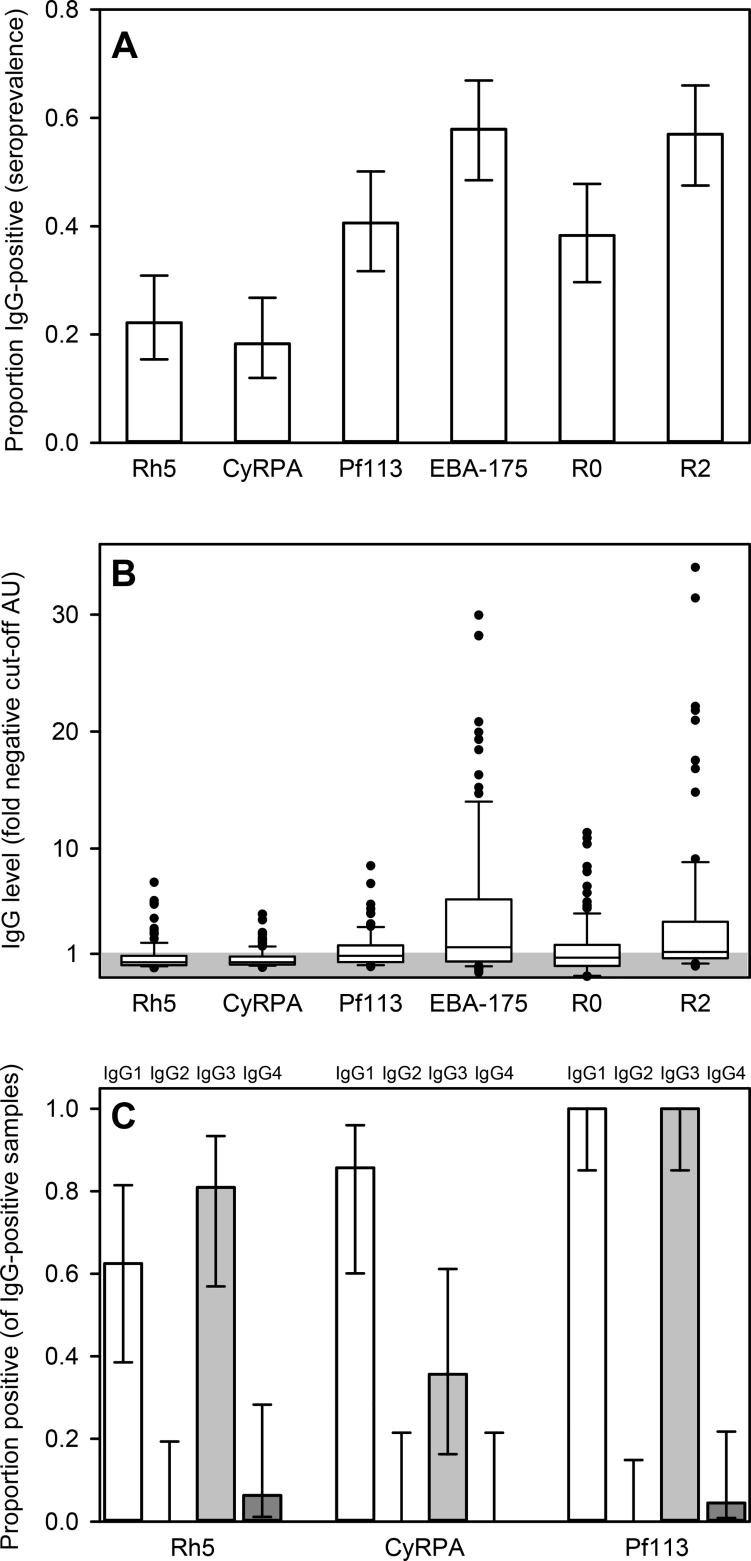
Merozoite-specific IgG in acutely ill *P*. *falciparum* malaria patients. A: Prevalences (proportions of donors with specific IgG levels above the negative cut-off) and their 95% confidence intervals (error bars) of merozoite-specific IgG in plasma of individual children with acute P. falciparum malaria. B: Levels of merozoite antigen-specific IgG in plasma, expressed as fold arbitrary units (AU) of the negative cut-off AU value for each antigen (indicated by the shaded area). Medians (center lines), central 50% (boxes), central 80% (bars), and outliers (dots) are indicated. C: Proportion of IgG-positive donors with detectable IgG subclass response to PfRH5 (left), CyRPA (center), and Pf113 (right). Proportions and corresponding 95% confidence intervals of IgG1 (white), IgG2 (black), IgG3 (gray), and IgG4 (dark gray) are shown. The presented data is from one experiment.

We first assessed the overall prevalence, levels and subclass composition of IgG specific for merozoite antigens in the plasma of the 118 children with confirmed *P*. *falciparum* malaria (Fig 1 and Table 1). The age of the children ranged from 1–12 years (Table 1).

**Table 1:** Clinical characteristics of study participants.

Category^1^	SM	UM	FC	AC	HC
Sex ratio (F/M)	14/28	33/33	1/9	15/13	31/29
Age (years)^2^	5(1 to 8)	4 (1 to 12)	5 (2 to 12)	6 (1 to 10)	5 (1 to 10)
Hemoglobin (g/dL)^2^	7.8 (4.1 to 11.8)	10.0 (5,4 to 14.2)	11.1 (6.3 to 13.0)	11.0 (6.5 to 12.8)	11.6 (4.2 to 15.0)
Parasitemia (/μL)^2^	52,200 (2,600–1,600,800)	40,200 (2900–649,000)	0	1,370 (300–1,900)	0

^1^SM (severe *P*. *falciparum* malaria), UM (uncomplicated *P*. *falciparum* malaria), FC (febrile controls), AC (asymptomatic controls), HC (healthy controls). Please refer to Materials and Methods for category definitions.

^2^Median (range)

Only about one in five of the patients had levels of PfRH5- and CyRPA-specific IgG above the negative cut-off (Fig 1A). One (PfRH5) or none (CyRPA) had high levels (>5× the negative cut-off) (Fig 1B). In marked contrast, more than half the study participants had levels of IgG above the negative cut-off to the well-studied merozoite antigens EBA175 and GLURP-R2 (Fig 1A), including many with high levels (28% and 21%, respectively) (Fig 1B). The prevalence of IgG specific for Pf113 and GLURP-R0 fell in between these extremes. The antibody responses to the PfRH5-complex antigens were completely dominated by IgG1 and IgG3 (Fig 1C). The IgG responses to PfRH5 and EBA175 did not correlate significantly (P>0.05) with the response to any of the remaining antigens but correlated with each other (P = 0.01) (Table 2), suggesting that antibody responses to PfRH5 and EBA175 are regulated differently from the other included merozoite antigens.

**Table 2:** Correlations (Spearman’s ρ [associated P-values] of IgG responses.

	CyRPA	Pf113	EBA175	GLURP-R0	GLURP-R2
PfRH5	0.16 [0.1]	0.08 [0.4]	0.24 [0.01]	0.10 [0.3]	0.12 [0.2]
CyRPA		0.62 [<0.001]	0.03 [0.8]	0.32 [0.001]	0.36 [<0.001]
Pf113			0.18 [0.06]	0.28 [0.004]	0.39 [<0.001]
EBA175				0.003 [1.0]	0.16 [0.1]
GLURP-R0					0.29 [0.002]

Overall, our findings correspond well with the limited published data on PfRH5 and Pf113 [22–26] which indicate that immune recognition of these antigens is poorer than other merozoite antigens such as EBA175 and GLURP (GLURP-R2, in particular) among individuals naturally exposed to *P*. *falciparum* [27, 28]. The observed dominance by cytophilic IgG sub-classes for all the antigens studied here is also in agreement with most previous studies of humoral immunity to *P*. *falciparum* asexual blood-stage antigens following natural exposure [19, 20, 23].

### The association with clinical presentation

**Fig 2:**
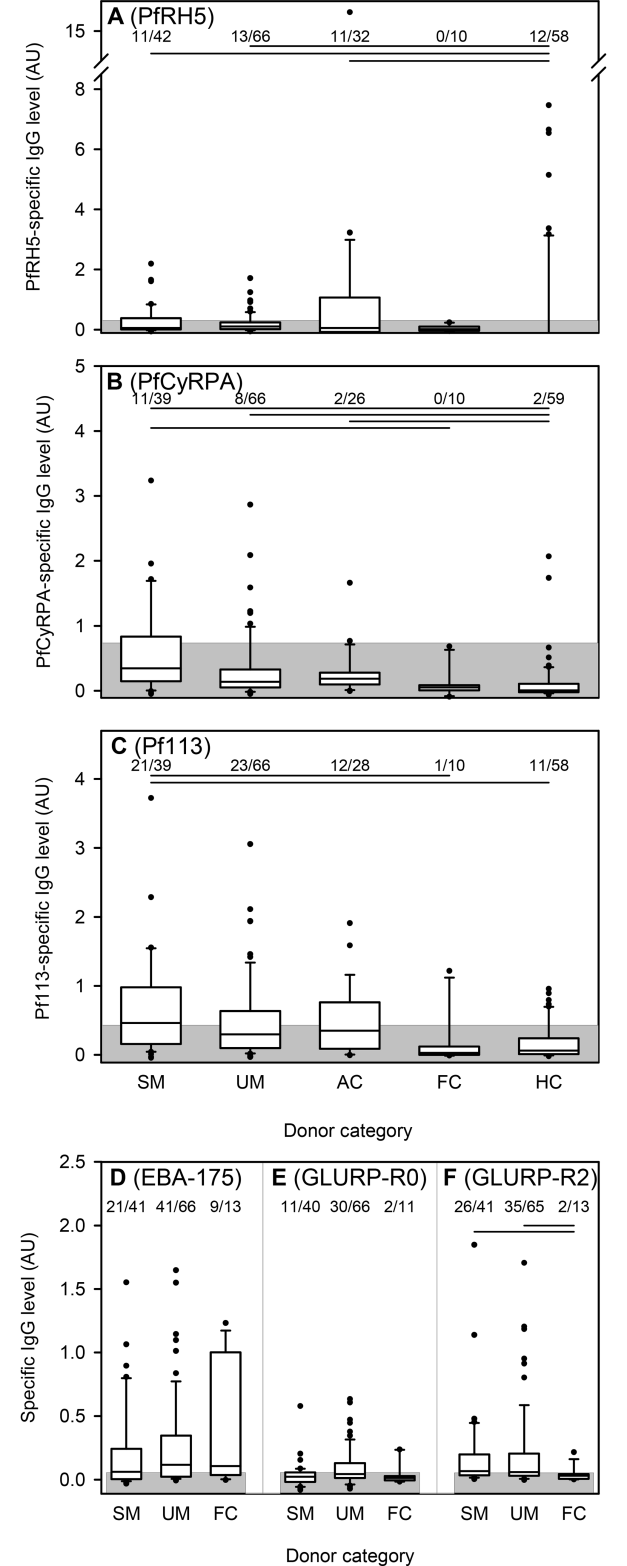
Merozoite-specific IgG according to clinical category. Levels (AU) of IgG specific for PfRH5 (A), CyRPA (B), Pf113 (C), EBA175 (D), GLURP-R0 (E) and GLURP-R2 (F) in plasma of individual children according to clinical category: SM (severe P. falciparum malaria), UM (uncomplicated P. falciparum malaria), FC (non-parasitemic febrile controls), AC (asymptomatic controls), HC (non-parasitemic healthy controls). Please refer to Materials and Methods for category definitions. The number of individuals with IgG above cut-off and the total number of individuals in each clinical category are given along the top of each panel. Horizontal lines along the top of the panels indicate statistically significant (P<0.05) differences between groups. Data presentation otherwise as in Fig 1B. The presented data is from one experiment.

The above data confirm that IgG with specificity for each of the studied merozoite antigens are induced to differing levels following natural exposure. Because IgG responses to several of them have been associated with protection following vaccination [18, 29] and considering the observed poor correlation among the IgG responses, we proceeded to sub-divide our study participants into distinct clinical categories, to assess the impact of recent parasite exposure on merozoite-specific IgG responses as well as their potential role as determinants of malaria susceptibility (Fig 2).

**Fig 3:**
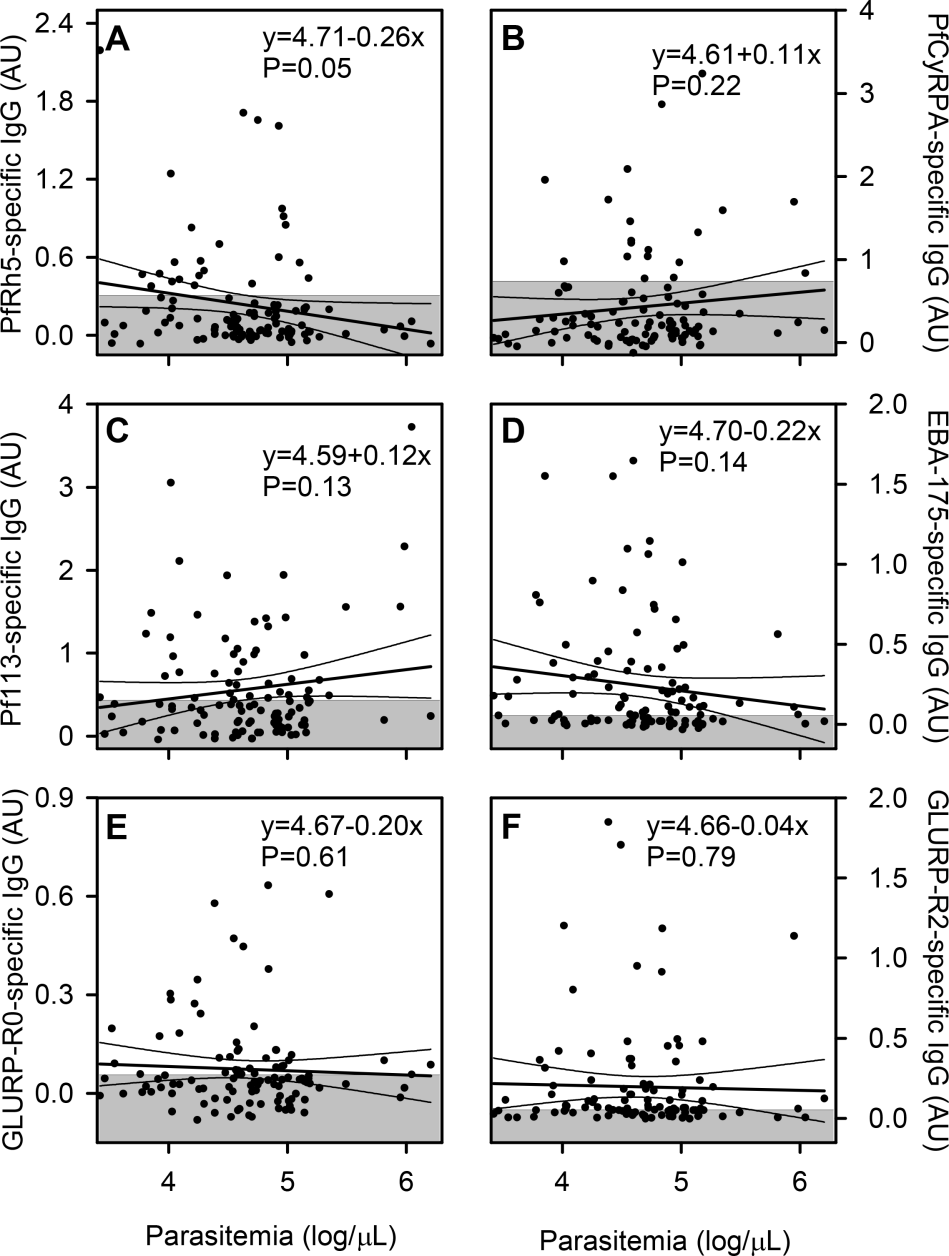
Relation between asexual *P*. *falciparum* parasitemia and merozoite-specific IgG. Correlations between parasitemia (parasites/μL) and plasma levels (AU) of IgG specific for PfRH5 (A), CyRPA (B), Pf113 (C), EBA175 (D), GLURP-R0 (E) and GLURP-R2 (F) in children with P. falciparum malaria. Data points for individual children, linear regression lines (and their 95% confidence intervals) for all the data, and negative cut-offs (shaded areas) are shown. The presented data is from one experiment.

The prevalence of positive IgG responses to PfRH5 did not differ significantly among the donor categories (P(χ^2^) = 0.24), although the proportion was somewhat higher (34%) among asymptomatically infected children (AC) than in the other categories (<27%) (Fig 2A). The median level was significantly lower in uninfected, healthy children (HC) than in AC children and children with uncomplicated (UM) or severe malaria (SM) (P<0.05, oneway- ANOVA on ranks followed by Dunn’s test). Thus, parasite exposure appears to boost PfRH5-specific IgG responses whether the infection causes symptoms or not. With respect to CyRPA, the proportion of responders did not differ significantly among the different donor categories, (P(χ^2^) = 0.24). Nonetheless, the proportion of responders in the SM group was higher (28%) than the other groups (<13%). The median IgG levels were significantly higher in the parasitemic donors than in the HC and FC donors (Fig 2B). For Pf113, the proportion of responders did not differ significantly (P(χ^2^) = 0.22) among the different donor categories. However, the median IgG levels was markedly higher in the severe malaria (SM) donors in comparison to the febrile (FC) and afebrile (HC) children without detectable parasitemia (P<0.05) (Fig 2C). For the control antigens EBA175 and GLURP-R0, proportions and levels did not differ significantly among the clinical groups (AC and HC donors were not tested due to lack of reagents) (Fig 2D and 2E). With GLURP-R2, there was a significant difference in the median IgG levels and the proportion of responders between the parasitemic donors (SM and UM) and the febrile donors (P<0.05). We next investigated the correlation between the antibody levels and parasitemia at enrolment and did not observe any statistically significant relationships between the level of IgG specific for any of the antigens and the presenting parasitemia of the donor (Fig 3).

While it is difficult to draw firm conclusions from these results, the data suggest that responses to each of the tested antigens indicate parasite exposure more than anything else.

### Kinetics of merozoite-specific IgG responses

**Fig 4:**
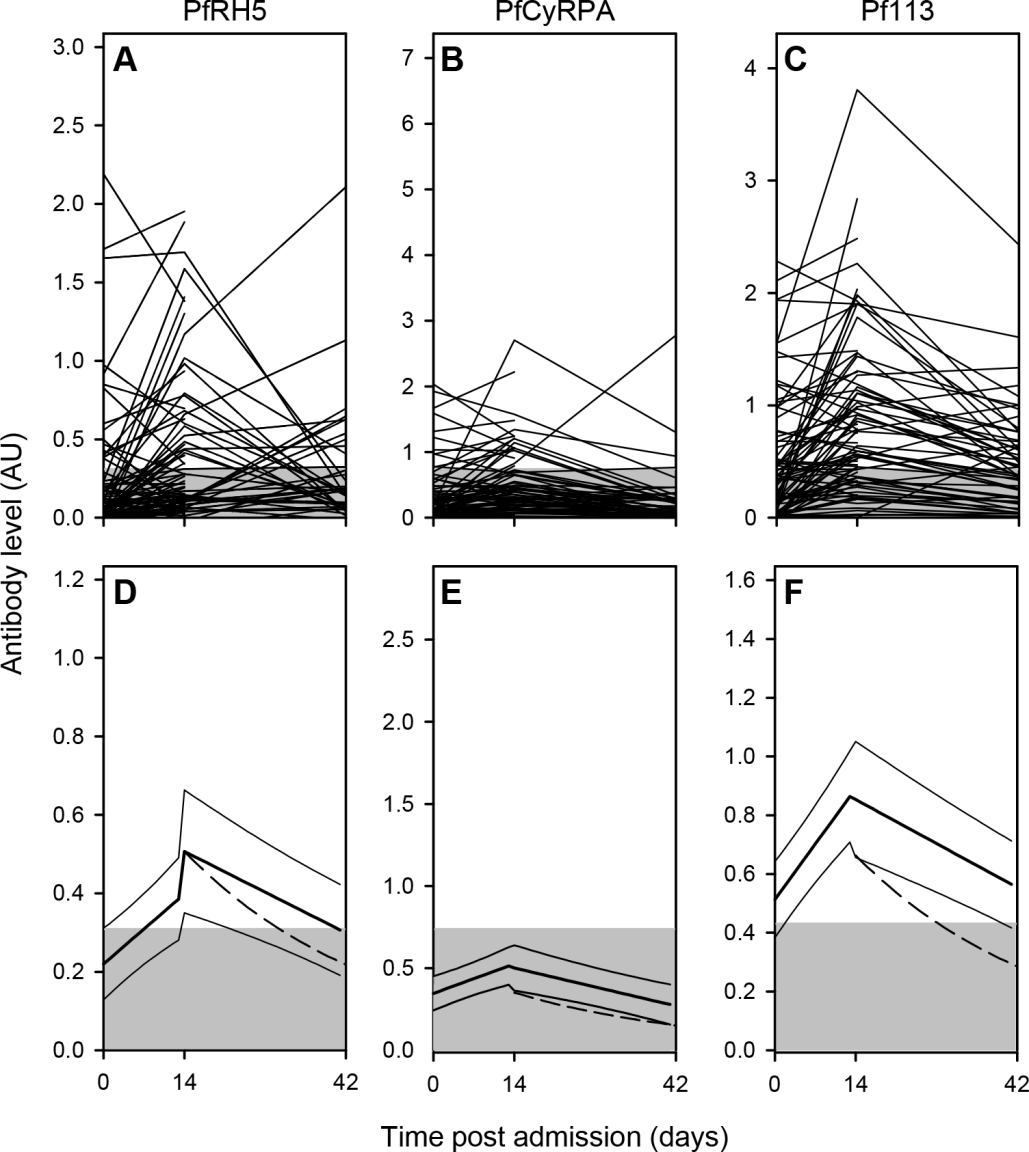
Kinetics of merozoite-specific IgG levels following episodes of *P*. *falciparum* malaria. Plasma levels of IgG specific for PfRH5 (A, D), CyRPA (B, E), and Pf113 (C, F) in children with P. falciparum malaria (Day 0), and in the same children two weeks (Day 14) and six weeks (Day 42) later. Temporal changes in levels of IgG in individual children (A-C) and in the cohort mean IgG level (D-F). Data from individual children are connected by lines (A-C). Cohort running means (heavy lines) and their 95% confidence intervals (thin lines), calculated as described previously [34], as well as calculated catabolic decay from Day 14 (dashed lines) are shown (D-F). Negative cut-offs (shaded areas) are shown (all panels). The presented data is from one experiment.

IgG responses to all the antigens except EBA175 tended to be higher in parasitemic children (donor categories SM, UM, AC) than in children without detectable parasitemia (donor categories FC and HC) (Fig 2). This suggests that IgG levels reflect recent exposure to *P*. *falciparum* and that the immune responses induced by infection are relatively transient. To obtain more direct information about the duration of the responses, we also measured IgG levels to the three PfRH5-complex antigens (PfRH5, PfCyRPA, and Pf113) in samples obtained two (Day 14) and six weeks (Day 42) after the initial diagnosis of *P*. *falciparum* malaria (Fig 4). In the majority of the children, the levels of IgG specific for each of the antigens were below the negative cut-off at admission and remained there during follow-up. A minority of children had IgG levels above cut-off on Day 14, but these had generally declined towards baseline by Day 42 (Fig 4A–4C) where the decline nearly followed the half-life of IgG (dotted line). Overall, responses to PfRH5, PfCyPA and Pf113 appeared to be short-lived (Fig 4D–4F), although malaria-induced Pf113 responses were most prevalent (Fig 4F).

### Assessment of clinically relevant PfRH5-specific IgG levels

Levels of IgG specific for several merozoite-specific antigens have been associated with clinical protection from *P*. *falciparum* malaria [30]. Furthermore, vaccination with PfRH5 constructs has been correlated with protection in experimental animals [18]. In the present study, we generally found low levels of such antibodies (Fig 1) and little evidence of an association with a better clinical outcome (Fig 2 and Fig 3). However, a few of the AC and HC donors examined had high levels of PfRH5-specific IgG (Fig 2A). We therefore tested a subset of our plasma samples in an additional PfRH5-specific standardized ELISA to be able to compare with values shown to be associated with effective merozoite invasion inhibition in an *in vitro* growth inhibition assay (GIA) and converting the AU into concentration of PfRH5 specific IgG [31]. Results from the two ELISA assays were very well correlated (P<0.0001; r = 0.92; N = 76) and converted values ranged from 5.5 ng/mL to 1.5μg/mL. The Rh5 specific IgG titres among our cohort were all below the 50% GIA cut-off (8.2μg/mL) observed in PfRH5 vaccinated volunteers [31] and therefore not sufficiently high to suggest ability to inhibit merozoite invasion by anti-PfRH5 antibodies only.

## Discussion

In this study, we analyze antibodies specific for three potential blood stage malaria vaccine candidates. It is the first longitudinal study comparing the acquisition and kinetics of naturally acquired antibodies towards PfRH5, Pf113 and CyRPA in the same cohort of children. Furthermore, it is the first study reporting on detectable levels of naturally acquired CyRPA antibodies. Several reports do exist on naturally acquired PfRH5-reactive antibodies [19–21, 32], including one longitudinal [22]. In the longitudinal study, PfRH5 specific antibody levels in Kenyan children were measured before and after the rainy season. By doing so, it was clear, that malaria exposure did boost the level of PfRH5 antibodies, but there was no follow up sample to determine the decay or maintenance of the antibody titres. Our results support their findings, since children admitted to hospital with malaria had higher levels of PfRH5 specific antibodies than the malaria negative controls. What is less clear in most reports on PfRH5 specific antibody levels is the association with protection against clinical malaria. Some studies have associated PfRH5 specific antibody levels with protection from malaria [20, 22]. In one study, only specific antibodies for both PfRH5 and Pf113 were analyzed [24], with a focus on differences in a rural and an urban area of Gabon. Their data from rural Gabon are comparable with our data from Hohoe, supporting a correlation between endemicity and PfRH5 and Pf113 antibody prevalence.

The prevalence of anti-Pf113 antibodies found in this study was slightly lower but comparable to levels observed in studies from Kenya and Gabon (50 and 51% respectively). This slight difference probably reflects differences in endemicity in the different areas or the age range of the children. In Imboumy-Limoukou et al, 2016 the most prevalent subclass for the Pf113 specific antibodies were IgG4, whereas we found IgG1 and IgG3 to be most abundant similarly to all other antigens tested here. That fact that IgG1 and IgG3 were the most abundant subclasses in our cohort points to a possibility of an antibody-mediated protection not only via blocking invasion, but also by compliment deposition and opsonization for phagocytosis.

The invasion complex antigens PfRH5 and CyRPA appeared to be less immunogenic than all the other studied merozoite surface antigens in this cohort of naturally exposed children with *P*. *falciparum* malaria. In other studies comparing the prevalence and levels of PfRH5 specific antibodies to other merozoite antigens such as Apical Membrane Antigen 1(AMA1) and Merozoite Surface Protein 1 (MSP1), the same pattern was seen [16, 19], but until now, not much has been reported for CyRPA specific antibodies. Whether the lower immunogenicity reflects direct properties of the proteins or the very short exposure time of the two complex antigens cannot be determined by these kinds of serological studies. In *Aotus* monkeys, vaccinations with both PfRH5 protein as well as viral vectored PfRH5 induced protective antibodies, supporting PfRH5 as a good immunogen [18]. A respectable antibody response was also seen after viral vectored administration of PfRH5 in humans (at much higher levels than those reported here), again favoring PfRH5 as an immunogenic protein following vaccination [31]. The discrepancy to natural infection could then be due to either competition with other immunogens during the infection, too short exposure time or a combination of the two.

In general, we observed a rapid reduction in antibody levels to the three studied antigens after malaria exposure. The malaria induced antibodies to PfRH5-complex proteins appears to be secreted by short lived plasma cells. But it is clear from our data, as well as the results of others [31], that the levels of PfRH5 found in naturally immune individuals are far too low to induce any real pronounced protection; moreover the same seems likely also to be the case with Pf113 and PfCyRPA antibodies, although these remain to be formally quantified. In contrast, vaccine induced antibodies against at least PfRH5 looks more promising in regards to the ability to reach protective levels and it would now be relevant to investigate vaccines combining PfRH5 proteins with other *P*. *falciparum* antigens.

## Materials and methods

### Study site and participants

The study was conducted in Hohoe, a town located about 220 km northeast of Accra, in an area of tropical semi-deciduous forest vegetation. Malaria transmission intensity in the area is high and has two seasonal peaks: a major one in April-July and a minor one in September-November. The dominant malaria species is *P*. *falciparum*, although cases of *P*. *ovale* and *P*. *malariae* infection are occasionally seen [33].

A total of 118 febrile (>37.5°C, present or within the last 24 h) children aged 1–12 years reporting to Hohoe Municipal Hospital on clinical suspicion of *P*. *falciparum* malaria were recruited to the study after informed consent had been obtained from a parent or legal guardian. Malaria patients (N = 108) were those where presence of *P*. *falciparum* infection was confirmed by a positive rapid diagnostic test (RDT) and by light microscopy with a parasite density > 2,500 parasites/μL. These malaria patients were subdivided into those with severe malaria (SM, N = 48) or uncomplicated malaria (UM, N = 60), respectively. SM was defined by the presence of one or more of the following without any other obvious unrelated cause: (i) impaired consciousness (Blantyre coma score ≤3) lasting longer than 30 minutes, (ii) acidosis, defined as respiratory distress (rapid, deep, and labored breathing), (iii) hypoglycemia (blood glucose <2.2 mM), (iv) hemoglobin concentration <5 g/dL, (v) >2 convulsions within the last 24 h, (vi) shock, and/or (vii) hyperparasitemia (>500,000/μL). Malaria patients without any of these SM criteria were classified as UM. Febrile patients without detectable parasitemia were grouped as febrile controls (FC, N = 10). All children were treated according to country guidelines. SM and UM children were seen again 14 (Day 14) and 42 days (Day 42) after the day of diagnosis (Day 0). In addition, 88 clinically healthy and age-matched children from within the Hohoe community were recruited in a similar manner, and grouped as either asymptomatic (AC, N = 28) or uninfected (HC, N = 60) controls. AC children were RDT-positive, and some had low parasitemia (<2,000/μL), whereas UC children were negative by microscopy and RDT. The main clinical characteristics of all the study participants are summarized in Table 1.

### Blood sample collection and research ethics clearance

One (in groups FC, AC, UC) or one to three venous blood samples (in groups SM, UM; on Day 0, Day 14, and Day 42) were collected in EDTA Vacutainers from each participant, in addition to the clinical parameters summarized in Table 1. Plasma was separated by centrifugation and stored at -20°C until use. The study was approved by the Ethics Committee of the Noguchi Memorial Institute for Medical Research, University of Ghana and by Ghana Health Service (GHS-ERC 08/05/14). Plasma samples from 10 non-exposed, anonymous Danish adults were used to establish the negative cut-off and to normalize ELISA data. The collection and use of those samples were approved by the Regional Research Ethics Committees for the Capital Region of Denmark (Protocol H-4-2013-083).

### *P*. *falciparum* merozoite antigens

Recombinant PfRH5 was expressed using *Drosophila melanogaster* Schneider 2 stable cell line system as described in detail previously [34] and used in a recent clinical trial ELISA [31]. CyRPA and full length ectodomain of Pf113 [9] were expressed by transient transfection of HEK293 cells as described in [35]. Recombinant GLURP antigens, representing the non-repetitive, amino-terminal domain R0 (amino acids 24–489) and the carboxy-terminal repeat region R2 (amino acids 705–1178), respectively, were expressed in *Escherichia coli* as described previously [36]. The receptor-binding domain of EBA175 was expressed as a recombinant protein in baculovirus-infected insect cells as described previously [37].

### Measurements of antigen-specific antibody levels in plasma

Plasma levels of IgG specific for PfRH5, GLURP-R0, GLURP-R2, and EBA175 were determined by ELISA as described for other malaria antigens in [38]. In summary, 96-well flat-bottomed plates were coated with recombinant antigen (2 μg/mL). After BSA-blocking and washing, plasma samples (1:100) were added in duplicate and incubated (1 h, room temp.). Unbound antibody was washed off and antigen-specific IgG detected by horseradish peroxidase-conjugated rabbit anti-human IgG (1:3,000) followed by O-phenylene diamine, and reading the optical density (OD) at 490 nm. Plasma samples from 10 Danish non-exposed individuals were used to establish the negative cut-off (mean+3 S.D.), while a plasma pool from semi-immune adults was used as a positive control and to normalize plate-to-plate variation. Plasma levels of IgG specific for CyRPA and Pf113 were measured by ELISA as described previously [17] and essentially as above, except for the blocking (casein, 1% w/v in PBS), and the detection system (alkaline phosphatase-conjugated goat anti-human IgG followed by p-nitrophenylphosphate and OD reading at 405 nm). A plasma pool from semi-immune adults was used as positive control and to normalize plate-to-plate variation. IgG Isotype ELISA was determined following standardized methodology described previously (Biswas et al, 2014). To convert the ELISA O.D values to ng/mL in predicting the GIA activity of the sera samples, standardized ELISA was performed as described in [31].

In all cases, antibody levels are presented as arbitrary units calculated as (OD_sample_−OD_blank_)/(OD_positive control_−OD_blank_). The cutoff for seropositivity was defined as normalized ELISA OD + (3 x mean standard deviations) of the non-exposed sera samples.

### Data analysis

One-way ANOVA and Dunn’s post-hoc test were used to evaluate differences in antibody levels among donor groups, while Mann-Whitney’s test was used to assess malaria severity-related differences in antibody levels. Pairwise associations between D0 antibody levels to different antigens, and between D0 antibody levels and log-transformed parasitemias were assessed by Spearman’s rank correlation test. Temporal changes in antibody levels were evaluated using the running means method described elsewhere [39].
