# Correction: Kinetics of antibody responses to PfRH5-complex antigens in Ghanaian children with *Plasmodium falciparum* malaria

**DOI:** 10.1371/journal.pone.0204452

**Published:** 2018-09-17

**Authors:** Frederica D. Partey, Filip C. Castberg, Edem W. Sarbah, Sarah E. Silk, Gordon A. Awandare, Simon J. Draper, Nicholas Opoku, Margaret Kweku, Michael F. Ofori, Lars Hviid, Lea Barfod

The affiliation for the eleventh author is incorrect. Lea Barfod is not affiliated with #6 but with #5 The Jenner Institute, University of Oxford, Oxford, United Kingdom.
